# S41. SHORT- AND LONG-TERM OUTCOMES OF VITAMIN D SUPPLEMENTATION IN CLOZAPINE TREATED CHRONIC SCHIZOPHRENIA PATIENTS

**DOI:** 10.1093/schbul/sby018.828

**Published:** 2018-04-01

**Authors:** Amir Krivoy, Roy Onn, Yael Vilner, Zvika Friedman, Abraham Weizman

**Affiliations:** Geha Metal Health Center

## Abstract

**Background:**

Vitamin D deficiency is highly prevalent in patients with psychosis. While accumulating data suggests that vitamin D deficiency may be involved in the clinical and metabolic outcomes of schizophrenia, there are no vitamin D supplementation studies in this context. Objective: To assess the short- and long-term impact of vitamin D supplementation on psychiatric and metabolic status in chronic clozapine-treated schizophrenia patients.

**Methods:**

Following a first phase of eight-week, randomized, double blind, placebo-controlled clinical trial, in which schizophrenia patients who had been maintained on clozapine treatment for at least 18 weeks with total PANSS scores >70 and with low levels of vitamin D (<75 nmol/L) were recruited, a second phase, post-RCT, was performed. In the RCT patients were randomly allocated to either weekly oral drops of vitamin D (14,000 IU) or placebo and subsequently assessed at two-week intervals regarding psychosis severity, mood, cognition and metabolic status. In the post-RCt phase, all participants were assessed at 24 weeks’ time point, while being prescribed 800 IU vitamin D daily supplementation in an open-label design.

**Results:**

Twenty-four patients were randomly assigned to vitamin D and the other 23 patients to the placebo arm. No between-group differences were found in baseline measures. After eight weeks, the vitamin D group had higher increase in vitamin D levels (31.4 vs -0.4 nM, p<0.0001). There was no significant effect of vitamin D on PANSS score, depression or metabolic parameters. The vitamin D group, however, showed a positive small effect on cognitive function (effect size=0.17). On the long-term follow up, 37 patients completed the 24-week assessment. Mean vitamin D levels did not change from baseline (57.56 nM to 57.28 nM) and no association was found between this change and the changes in psychiatric, metabolic and cognitive measures. The only significant inverse association was found between vitamin D levels and HDL (p=0.007).

**Discussion:**

This is the first study to assess the outcomes of vitamin D as an adjunct to clozapine in chronic schizophrenia patients, in the short and long term. The increase in vitamin D levels on the short-term was associated with a small positive effect on cognition, without any effect on psychosis, mood or metabolic status. There were no significant effects of vitamin D supplementation on the assessed measures in the long-term either.

